# Postpartum Glucose Tolerance Testing Among Patients With Gestational Diabetes During the Coronavirus Disease 2019 Pandemic

**DOI:** 10.7759/cureus.34210

**Published:** 2023-01-25

**Authors:** Ariane C Youssefzadeh, Laurel S Aberle, Brian Gordon, Intira Sriprasert, David A Sacks, Bhuvan Martin, Paola Sequeira, Richard H Lee

**Affiliations:** 1 Department of Obstetrics and Gynecology, Los Angeles County University of Southern California Medical Center, Los Angeles, USA; 2 Department of Obstetrics and Gynecology, Huntington Hospital, Pasadena, USA; 3 Department of Endocrinology, Los Angeles County University of Southern California Medical Center, Los Angeles, USA

**Keywords:** types 2 diabetes, postpartum care, covid-19, postpartum glucose tolerance testing, gestational diabetes

## Abstract

Introduction

The impact of the coronavirus disease (COVID-19) COVID-19 pandemic on the care of pregnant patients with gestational diabetes (GDM) is largely unreported. The objective of this study was to compare the completion of postpartum oral glucose tolerance testing (GTT) prior to and during the COVID-19 pandemic among patients with GDM.

Methods

This was a retrospective review of patients diagnosed with GDM between April 2019 and March 2021. Medical records of patients diagnosed with GDM prior to and during the pandemic were compared. The primary outcome was the difference in the completion of postpartum GTT prior to and during the COVID-19 pandemic. Completion was defined as testing between four weeks to six months postpartum. Secondary objectives were: 1) to compare maternal and neonatal outcomes prior to and during the pandemic among patients with GDM, and 2) to compare pregnancy characteristics and outcomes by compliance with postpartum GTT.

Results

There were 185 patients included in the study, of whom 83 (44.9%) delivered prior to the pandemic and 102 (55.1%) delivered during the pandemic. There was no difference in completion of postpartum diabetes testing prior, compared to during the pandemic (27.7% vs 33.3%, p=0.47). Postpartum diagnosis of pre-diabetes and type two diabetes mellitus (T2DM) did not differ between groups (p=0.36 and p=1.00, respectively). Patients who completed postpartum testing were less likely to have preeclampsia with severe features compared to patients who did not (OR 0.08, 95% CI 0.01-0.96, p=0.02).

Conclusion

Completion of postpartum testing for T2DM remained poor prior to and during the COVID-19 pandemic. These findings underscore the need for the adoption of more accessible methods of postpartum testing for T2DM among patients with GDM.

## Introduction

Patients with a history of gestational diabetes (GDM) carry a 10-fold increased risk of developing type two diabetes mellitus (T2DM) in their lifetime [1]. Postpartum testing for diabetes is recommended for all patients diagnosed with GDM by The American College of Obstetricians and Gynecologists (ACOG) [2] and by the American Diabetes Association (ADA) [3]. However, the majority of women with gestational diabetes do not undergo the recommended postpartum testing [4-12].

Testing for diabetes is recommended four to 12 weeks after delivery using a 75-gram two-hour glucose tolerance test (GTT) [2,3]. However, due to poor compliance with the standard two-hour GTT, investigators have explored more tolerable and less time-consuming alternatives such as glycosylated hemoglobin (HbA1C) or fasting blood glucose [5,13]. More convenient timing of testing relative to delivery has also been explored, including shifting testing to the immediate postpartum period via a 24-72 hour postpartum fasting glucose or a postpartum day two GTT [4,13,14].

Since the onset of the coronavirus disease 2019 (COVID-19) pandemic, patient care has rapidly evolved to meet the needs of a burdened healthcare system while maintaining social distancing practices. Some have advised delaying the postpartum GTT or simplifying postpartum testing with an HbA1C test among patients with gestational diabetes during the pandemic [15]. However, overall, the emphasis in the existing literature is on alternative strategies to the antepartum GTT with less focus on the postpartum GTT [15, 16]. Thus, the primary objective of this study was to assess the effect of the COVID-19 pandemic on patients' completion of the postpartum GTT. Elements of this study were previously presented during a poster presentation at the 42nd Annual Meeting of the Society for Maternal-Fetal Medicine on February 4, 2022.

## Materials and methods

This is a retrospective observational study of postpartum testing for T2DM among patients with GDM. Electronic medical records were reviewed for all deliveries at a single institution, Los Angeles County + University of Southern California (LAC+USC) Medical Center in Los Angeles, between April 1st, 2019, and March 31st, 2021. Patients with singleton pregnancies complicated by GDM were included in the study. At our institution, GDM is diagnosed using ACOG's recommended two-step protocol [2]. Patients were excluded if they had pre-existing type one or two diabetes, multifetal gestation, or if providers failed to order the postpartum 75-gram two-hour GTT. The study was reviewed by the institutional review board and considered to be exempt.

The primary objective of this study was to compare the proportion of patients who completed the recommended postpartum GTT prior to versus during the COVID-19 pandemic. To evaluate this objective, patients who delivered during the study interval were divided into two groups based on the date of delivery. COVID-19 was declared a pandemic by the World Health Organization (WHO) on March 11th, 2020 [17]. So, for the purposes of this study, patients who delivered prior to March 1st, 2020, were considered prior to the COVID-19 pandemic. Completion of postpartum testing follow-up was defined as obtaining the 75-gram two-hour GTT from four weeks to six months postpartum.

Secondary objectives were 1) to compare maternal and neonatal outcomes prior to and during the COVID-19 pandemic among patients with GDM, and 2) to compare patient and pregnancy characteristics between patients who did and did not complete postpartum testing across the entire study interval. Patient demographics, pregnancy characteristics, maternal outcomes, and neonatal outcomes were collected by review of electronic medical records.

Diagnosis of prediabetes and T2DM were according to the American Diabetes Association (ADA) [18]. Normal glucose tolerance was defined as fasting blood glucose <100 mg/dl and <140 mg/dl two hours following a 75-gram glucose load. Prediabetes was defined as fasting blood glucose of 100-125 mg/dl or blood glucose of 140-199 mg/dl two hours following a 75-gram. Finally, T2DM was defined as a fasting plasma glucose of ≥126 mg/dl or a plasma glucose of ≥200 mg/dl two hours following a 75-gram glucose load [3]. Venous plasma glucose samples were analyzed with a hexokinase assay (Cobas Model 4000; Roche Diagnostics, Indianapolis, Indiana).

A sample size of at least 80 per group (prior to and during the COVID-19 pandemic), of which 30% of patients completed postpartum GTT, could detect at least a 22% difference in postpartum GTT completion with 80% power and an alpha of 0.05. Continuous variables were reported as mean (standard deviation) or median (interquartile range), and compared using student t-test or Wilcoxon rank sum test, respectively. Categorical variables were reported as frequency (%) and compared with the Chi-squared test. Multivariate logistic regression analysis controlling for age, BMI, and parity tested the association between postpartum diabetes testing the completion prior to and during the COVID-19 pandemic. The statistical analysis was performed with Stata software (StataCorp, College Station, Texas).

## Results

During the study interval, 220 patients with GDM delivered at our institution. Among those patients, 35 patients were excluded because the postpartum GTT was not ordered (n=28, 12.7%) and/or the pregnancy was a multifetal gestation (n=8, 3.6%). In total, 185 patients met the inclusion criteria. Among these patients, 83 (44.9%) delivered prior to the pandemic, and 102 (55.1%) delivered during the pandemic.

Baseline characteristics did not differ during both periods (Table 1). The proportion of patients who completed the postpartum GTT did not differ prior to and during the pandemic. This finding was unchanged after multivariate logistic regression when controlling for age, body mass index (BMI), and parity (OR 1.11, 95% CI 0.58-2.15, p=0.75). Most maternal and neonatal outcomes did not differ between the two cohorts. Preeclampsia without severe features occurred more frequently among women who had pregnancies complicated by GDM before the pandemic than among those who had GDM during the pandemic (15.7% versus 1.0%, p<0.001). This finding remained significant after multivariate logistic regression when controlling for age, BMI, and parity (OR=0.06, 95% CI 0.01-0.46, p=0.01).

**Table 1:** Baseline characteristics and pregnancy outcomes of patients with GDM who delivered before and during the COVID-19 pandemic Results are expressed as n (%), mean ± standard deviation, or median (25th quartile - 75th quartile) *Body mass index at time of initial presentation during index pregnancy †Data missing for three patients pre-pandemic and nine patients during the pandemic ‡From gestational age on presentation to delivery §Data missing for one pre-pandemic patient and one patient during the pandemic GDM - gestational diabetes, GTT - glucose tolerance testing

Category	Prior to the pandemic (n= 83)	During pandemic (n= 102)	p-value
Completion of 75-gram GTT 4 weeks - 6 months postpartum	23 (27.7%)	34 (33.3%)	0.43
Age (years)	31.8 ± 5.7	32.4 ± 6.0	0.47
*Body mass index (kg/m^2^)	33.5 ± 7.5	31.6 ± 7.5	0.07
Parity	2 (1-3)	2 (1-2)	0.69
Race/ethnicity			0.28
Asian	2 (2.4%)	9 (8.8%)	
Black	4 (4.8%)	5 (4.9%)	
Hispanic	65 (78.3%)	78 (76.5%)	
Non-Hispanic white	1 (1.2%)	0	
None of the above	11 (13.3%)	10 (9.8%)	
Chronic hypertension	9 (10.8%)	9 (8.8%)	0.65
History of cesarean section	16 (19.3%)	25 (24.5%)	0.48
GDM in prior pregnancy	16 (19.3%)	15 (14.7%)	0.43
^†^Gestational age at GDM diagnosis (weeks)	23.8 ± 8.7	24.1 ± 8.4	0.78
Diet-controlled GDM	51 (61.5%)	73 (71.6%)	0.16
^‡^Maternal weight gain (kilograms)	5.2 ± 6.0	6.5 ± 6.0	0.16
Gestational age at delivery (weeks)	38.1 ± 2.2	38.2 ± 2.3	0.94
^§^Infant birth weight (grams)	3211 ± 682	3216 ± 666	0.96
Gestational hypertension	4 (4.8%)	4 (3.9%)	1.00
Preeclampsia without severe features	13 (15.7%)	1 (1.0%)	0.00
Preeclampsia with severe features	5 (6.0%)	13 (12.7%)	0.14
Cesarean section (total)	28 (33.7%)	46 (45.1%)	0.13
Scheduled	14 (16.9%)	20 (19.6%)	0.26
Shoulder dystocia	2 (2.4%)	2 (2.0%)	1.00
Postpartum hemorrhage	13 (15.7%)	16 (15.7%)	1.00
Chorioamnionitis	8 (9.6%)	11 (10.8%)	1.00
Wound complication	1 (1.2%)	4 (3.9%)	0.35
Neonatal intensive care unit admission	26 (31.3%)	33 (34.4%)	1.00

Patients who completed postpartum testing were older and had a lower BMI at the time of presentation to our institution compared with patients who did not complete the postpartum GTT (Table 2). Maternal and neonatal outcomes were similar between patients who did and did not complete with testing, except that preeclampsia with severe features was more common among patients who did not complete postpartum testing (multivariate logistic regression analysis, OR= 0.08, 95% CI 0.01-0.96, p=0.02).

**Table 2:** Baseline characteristics and pregnancy outcomes of patients with GDM who did and did not complete postpartum glucose tolerance testing (GTT) Results are expressed as n (%), mean ± standard deviation, or median (25th quartile - 75th quartile) *Body mass index at time of initial presentation during index pregnancy †Data missing for 11 patients who were not compliant and one patient who was compliant with postpartum GTT ‡Data missing for one patient who did not complete postpartum GTT GDM - gestational diabetes, GTT - glucose tolerance testing

Category	Did not complete GTT (n=128)	Completed GTT (n=57)	p-value
Age (years)	31.5 ± 6.0	33.4 ± 5.4	0.04
^*^Body mass index (kg/m^2^)	33.5 ± 7.7	30.1 ± 6.1	<0.001
Parity	1 (1-3)	2 (1-2)	0.88
Race/ethnicity			0.15
Asian	5 (3.9%)	6 (10.5%)	
Black	7 (5.5%)	2 (3.5%)	
Hispanic	97 (75.8%)	46 (80.7%)	
Non-Hispanic White	1 (0.8%)	0	
None of the above	18 (14.1%)	3 (5.3%)	
Chronic hypertension	12 (9.4%)	6 (10.5%)	0.81
History of cesarean section	26 (20.3%)	15 (26.3%)	0.44
History of GDM	23 (18.0%)	8 (14.0%)	0.67
^†^Gestational age at GDM diagnosis	24.6 ± 8.6	22.7 ± 8.2	0.18
Diet-controlled GDM	86 (67.2%)	38 (66.7%)	1.00
Gestational age at delivery (weeks)	38.2 ± 2.2	38.1 ± 2.2	0.80
^‡^Infant birthweight (grams)	3279 ± 683	3069 ± 624	0.05
Gestational hypertension	7 (5.5%)	1 (1.8%)	0.44
Preeclampsia without severe features	12 (9.4%)	2 (3.5%)	0.23
Preeclampsia with severe features	17 (13.3%)	1 (1.8%)	0.01
Cesarean delivery	50 (39.1%)	24 (42.1%)	0.75
Scheduled	21 (16.4%)	13 (22.8%)	0.57
Shoulder dystocia	3 (2.3%)	1 (1.8%)	1.00
Postpartum hemorrhage	22 (17.2%)	7 (12.3%)	0.51
Chorioamnionitis	12 (9.4%)	7 (12.3%)	0.60
Wound complication	4 (3.1%)	1 (1.8%)	0.61
Neonatal intensive care unit admission	39 (30.5%)	20 (35.1%)	0.61

In total, 17 (29.8%) of the 57 patients who obtained the recommended GTT had abnormal results. Of these 17 patients, 10 (58.8%) had antenatally diet-controlled GDM. One of the 57 patients did not complete fasting glucose at the time of the two-hour GTT blood test though her two-hour value was in the pre-diabetes range. Otherwise, this patient’s data were excluded from further analysis (Table 3). Results of the GTT did not differ between the two cohorts.

**Table 3:** Results of the postpartum glucose tolerance test Results are expressed as n (%). *One patient with an abnormal test result (blood glucose 148 mg/dl two hours after 75-grams glucose load) was excluded from this analysis because fasting blood glucose was not recorded on the same day.

	Prior to the pandemic (n=22)	During the pandemic (n=34)	p
Abnormal	*8 (34.8%)	8 (23.5%)	0.37
Results consistent with pre-diabetes	7 (31.8%)	7 (20.6%)	0.36
Results consistent with type 2 diabetes mellitus	1 (4.5%)	1 (2.9%)	1.00

## Discussion

There are two key findings: first, most patients with GDM did not complete recommended postpartum testing for T2DM. Second, there was no difference in the rates of completion of the postpartum GTT before and during the COVID-19 pandemic.

Prior to this study, little was known about the impact of COVID-19 on postpartum testing for T2DM among GDM patients. There is a myriad of reasons one would expect compliance with postpartum diabetes testing to be lower during the pandemic, such as fear of exposure to COVID-19 in the healthcare setting and increased financial barriers affecting access to transportation, childcare, or the ability to miss work. However, the dismal rates of completion were stable over the study period. Thus, although the current clinical approach to postpartum diabetes testing did not worsen during the pandemic, change is still needed to improve rates of postpartum testing for diabetes. For example, evidence suggests greater patient adherence and comparable diagnostic ability of a GTT on postpartum day two compared to outpatient testing at four to 12 weeks postpartum [4,14]. 

In our study, patient characteristics and pregnancy outcomes were overall similar between patients who were and were not compliant with postpartum testing. Education level was not assessed here, though, in prior studies, this was an important risk factor for non-compliance with the recommended postpartum GTT [10,19]. Furthermore, there are other factors that may predict compliance with postpartum GTT not assessed here, such as the number of prenatal visits attended, availability of childcare, available leave time from work to get testing, distance from patient's home to the location of postpartum GTT site, and transportation. One notable difference is the higher rate of preeclampsia with severe features among patients who were not compliant with postpartum testing for T2DM. It is possible these patients were exposed to abbreviated or no counseling about the need for postpartum testing because attention was directed toward their more acute issue of severe hypertensive disease.

Here, most patients with abnormal postpartum GTTs had pre-diabetes rather than T2DM. This highlights the importance of postpartum testing because pre-diabetes is a potentially clinically reversible disease when appropriate interventions are offered [20,21]. Additionally, in this study, a large portion of patients with abnormal two-hour GTT results had diet-controlled GDM, which underscores the need for postpartum GTT for all patients with GDM.

Strengths of this study include a direct review of electronic medical records that ensured the accuracy of the variables analyzed. Limitations of this study include its modest sample size from a single institution and retrospective design, which reduced the ability to make causal inferences. In the absence of patient interviews or surveys, it is also unknown if any patients who delivered at our institution ultimately performed postpartum testing via an outside provider.

## Conclusions

In conclusion, completion of postpartum GTT among patients with pregnancies complicated by GDM remained equally poor both prior to and during the COVID-19 pandemic. However, given the small sample size in the present study, larger studies are needed to clarify the impact of the COVID-19 pandemic on postpartum GTT completion. Nonetheless, the findings here may improve patient care in that they highlight the urgent need for more accessible postpartum testing, regardless of the status of the pandemic. Current literature suggests inpatient postpartum day two GTT may be a viable alternative to later outpatient testing without compromising accurate diagnosis of T2DM, though future studies are needed to clarify the best alternative approach.

## References

[1] Vounzoulaki E, Khunti K, Abner SC, Tan BK, Davies MJ, Gillies CL (2020). Progression to type 2 diabetes in women with a known history of gestational diabetes: systematic review and meta-analysis. BMJ.

[2] (2018). ACOG practice bulletin no. 190 summary: gestational diabetes mellitus. Obstet Gynecol.

[3] Draznin B, Aroda VR, Bakris G (2022). Management of diabetes in pregnancy: standards of medical care in diabetes-2022. Diabetes Care.

[4] Carter EB, Martin S, Temming LA, Colditz GA, Macones GA, Tuuli MG (2018). Early versus 6-12 week postpartum glucose tolerance testing for women with gestational diabetes. J Perinatol.

[5] Carson MP, Ananth CV, Gyamfi-Bannerman C, Smulian J, Wapner RJ (2018). Postpartum testing to detect persistent dysglycemia in women with gestational diabetes mellitus. Obstet Gynecol.

[6] Rosenbloom JI, Blanchard MH (2018). Compliance with postpartum diabetes screening recommendations for patients with gestational diabetes. J Womens Health (Larchmt).

[7] Leuridan L, Wens J, Devlieger R, Verhaeghe J, Mathieu C, Benhalima K (2015). Glucose intolerance in early postpartum in women with gestational diabetes: Who is at increased risk?. Prim Care Diabetes.

[8] Tovar A, Chasan-Taber L, Eggleston E, Oken E (2011). Postpartum screening for diabetes among women with a history of gestational diabetes mellitus. Prev Chronic Dis.

[9] Aziz S, Munim TF, Fatima SS (2018). Post-partum follow-up of women with gestational diabetes mellitus: effectiveness, determinants, and barriers. J Matern Fetal Neonatal Med.

[10] de Gennaro G, Bianchi C, Aragona M (2020). Postpartum screening for type 2 diabetes mellitus in women with gestational diabetes: is it really performed?. Diabetes Res Clin Pract.

[11] Chang Y, Chen X, Cui H, Zhang Z, Cheng L (2014). Follow-up of postpartum women with gestational diabetes mellitus (GDM). Diabetes Res Clin Pract.

[12] Nouhjah S, Shahbazian H, Amoori N, Jahanfar S, Shahbazian N, Jahanshahi A, Cheraghian B (2017). Postpartum screening practices, progression to abnormal glucose tolerance and its related risk factors in Asian women with a known history of gestational diabetes: a systematic review and meta-analysis. Diabetes Metab Syndr.

[13] Duke A, Yap C, Bradbury R, Hng TM, Kim C, Wansbrough A, Cheung NW (2015). The discordance between HbA1c and glucose tolerance testing for the postpartum exclusion of diabetes following gestational diabetes. Diabetes Res Clin Pract.

[14] Werner EF, Has P, Rouse D, Clark MA (2020). Two-day postpartum compared with 4- to 12-week postpartum glucose tolerance testing for women with gestational diabetes. Am J Obstet Gynecol.

[15] Panaitescu AM, Ciobanu AM, Popa M, Duta I, Gica N, Peltecu G, Veduta A (2021). Screening for Gestational Diabetes during the COVID-19 Pandemic-Current Recommendations and Their Consequences. Medicina (Kaunas).

[16] Zhu S, Meehan T, Veerasingham M, Sivanesan K (2021). COVID-19 pandemic gestational diabetes screening guidelines: a retrospective study in Australian women. Diabetes Metab Syndr.

[17] (2020). Declaring a national emergency Concerning the novel coronavirus disease (COVID-19) outbreak. https://www.federalregister.gov/documents/2020/03/18/2020-05794/declaring-a-national-emergency-concerning-the-novel-coronavirus-disease-covid-19-outbreak..

[18] American Diabetes Association (2019). Classification and diagnosis of diabetes: standards of medical care in diabetes-2019. Diabetes Care.

[19] Fabiyi CA, Reid LD, Mistry KB (2019). Postpartum health care use after gestational diabetes and hypertensive disorders of pregnancy. J Womens Health (Larchmt).

[20] Hostalek U, Campbell I (2021). Metformin for diabetes prevention: update of the evidence base. Curr Med Res Opin.

[21] Knowler WC, Barrett-Connor E, Fowler SE, Hamman RF, Lachin JM, Walker EA, Nathan DM (2002). Reduction in the incidence of type 2 diabetes with lifestyle intervention or metformin. N Engl J Med.

